# Phytase Supplementation under Commercially Intensive Rearing Conditions: Impacts on Nile Tilapia Growth Performance and Nutrient Digestibility

**DOI:** 10.3390/ani13010136

**Published:** 2022-12-29

**Authors:** Edgar Junio Damasceno Rodrigues, Paulo Incane Ito, Lucas Franco Miranda Ribeiro, Pedro Luiz Pucci Figueiredo de Carvalho, William dos Santos Xavier, Matheus Gardim Guimarães, Ademir Calvo Fernandes Junior, Luiz Edivaldo Pezzato, Margarida Maria Barros

**Affiliations:** 1 School of Veterinary Medicine and Animal Science, Department of Breeding and Animal Nutrition, UNESP-São Paulo State University, Botucatu 18610-034, SP, Brazil; 2 Department of Ecology and Conservation Biology, Texas A&M AgriLife Research, Texas A&M University, College Station, TX 77845, USA

**Keywords:** floating cages, feed additive, *Oreochromis niloticus*, digestibility, fish farming

## Abstract

**Simple Summary:**

Fish farming is a fast-growing feed production sector that is expected to significantly contribute to world food safety. The awareness of the environmental impacts of this activity is increasing, as are the practices to enhance fish farming sustainability. Phytase is a feed additive that improves not only fish growth performance but also fish farming sustainability by reducing nutrient discharge in the water. In this regard, we evaluated phytase effects on nutrient digestibility and growth performance of Nile tilapia under commercially intensive rearing conditions. The results of this study demonstrate that phytase can be used as an approach to minimize fish farming environmental impacts and boost fish growth. Besides that, our results validate the findings of previous studies conducted under controlled laboratory conditions.

**Abstract:**

This study evaluated the effects of phytase supplementation on growth performance and apparent digestibility of Nile tilapia (*Oreochromis niloticus*) in a commercial fish farm setting. Nile tilapia (6300 male, 57.48 ± 1.04 g) were randomly stocked into 42 floating cages. The experimental design was completely randomized, comprising six treatments and seven replications. Fish were fed five phosphorus deficient plant-based diets with graded levels of phytase supplementation (0, 500, 1000, 1500, 2000 UF kg^−1^) and an additional diet containing phosphorus supplementation to meet the requirement of this fish species (positive control). After 97 days of feeding, growth performance data were collected and 900 fish (500 ± 10 g) were relocated to 6 floating cages for the digestibility assessment. Quadratic polynomial regression analysis indicated 1537.5 and 1593.2 UF kg^−1^ as the optimum dietary levels for daily weight gain and feed conversion rate, respectively. Including 2000 UF kg^−1^ resulted in the higher dry matter, crude protein, energy, and ash apparent digestibility coefficient values. Therefore, phytase supplementation from 1500 to 2000 UF kg^−1^ is recommended to enhance growth performance and nutrient bioavailability of Nile tilapia reared according to industry practices.

## 1. Introduction

Phytase is one of the most studied and supplemented enzymes in animal nutrition, being widely explored in poultry and swine production, and by the aquaculture industry to a lesser extent. However, even with the considerable utilization of phytase, there is a lack of studies evaluating its effects on Nile tilapia growth performance and nutrient availability under commercially intensive rearing conditions. Since most of the studies evaluating the positive effects of this enzyme on fish growth and nutrient utilization were conducted under controlled laboratory conditions as compiled by [1,2], it becomes increasingly important to compare the current data with data obtained under more practical and applicable settings. Nile tilapia is the third most cultivated species in the world aquaculture, with an 8.3% share [3], and the most cultivated species in Brazil, representing 61.21% of the total amount produced in 2020 [4]. In Brazil, floating net cage production became the most popular system for rearing tilapia due to productive advantages such as suitable water quality promoted by high flushing rates and water depth [5]. However, as this activity uses public water reservoirs its environmental impacts need to be restrained. In fact, fish farming in floating net cages may promote water course contamination with sediments, resulting in eutrophication and anoxic conditions [6].

This issue may be aggravated by the increase in plant feed ingredients used in fish diets. Even though plant byproducts are sustainable ingredients, there are several anti-nutritional factors in their composition that may reduce nutrient bioavailability, affect growth performance and animal metabolism, increasing discharges of nitrogen and other nutrients in the aquatic environment [7]. Phytate is one of those antinutrients, being found in cereals and legumes commonly used as feed ingredients in aquafeeds. This molecule is the phosphorus storage form of nuts, oil seeds, and cereal grains, which is not bioavailable for non-ruminant animals, including most fish species due to a lack of sufficient endogenous phytase [8,9,10]. The antinutritional capacity of phytate is associated with its negative charges which occur over a wide pH range and enables the interaction of this molecule with lipids, proteins, and carbohydrates. These interactions may considerably reduce the activity of enzymes, as well as the bioavailability and digestibility of dietary components [1].

A possible strategy to counterbalance the negative effects of phytate content in plant ingredients is including exogenous phytase in fish diets. Phytase is a phosphatase enzyme that catalyzes the sequential hydrolysis of phytate resulting in available inorganic phosphorus and inositol esters [1]. Indeed, using phytase as a feed additive can reduce the amount of inorganic phosphorus supplementation and decrease phosphorus and nitrogen excretion in feces, benefiting both animals and the environment. Under laboratory conditions, phytase supplementation resulted in a decrease of up to 56% in P [9] and 13% in total N waste loading [11]. This strategy is important since aquaculture production generated 0.46 million metric tons of phosphorus discharged in the aquatic environment in 2008 [12] and fish P retention remains around 40% in modern commercial fish farms, which is still far from being considered sustainable [13].

Additionally, phytase supplementation potentially improves the dietary bioavailability of nutrients and energy as well as feed efficiency and growth performance parameters, as demonstrated in laboratory studies. It was verified that supplementing 660 UF kg^−1^ in plant-based diets of Nile tilapia juveniles determined positive effects on growth performance and bioavailability of dry matter, energy, and starch [14]. Nutrient digestibility and growth performance parameters were also improved by supplementing 1000 UF kg^−1^ in juvenile Nile tilapia plant-based diets [15]. Higher dietary doses of phytase also improved nutrient and energy digestibility in juvenile Nile tilapia diets. Indeed, the supplementation of 1500 UF kg^−1^ increased the digestibility of crude energy, protein, and lipids, as well as P, calcium, and other minerals [16]. Ref. [17] verified that supplementing 2000 UF kg^−1^ in Nile tilapia-fed plant-based diets resulted in higher weight gain, nutrient, and energy digestibility, and utilization compared to the control diet. Moreover, the effects of phytase supplementation in fish diets regarding growth performance parameters, nutrient availability, and utilization were widely described in previous publications [1,2].

Although the effects of phytase on fish growth performance and nutrient digestibility are well described, to the authors knowledge there is no study assessing the effects of this feed additive in tilapia culture under practical rearing conditions. Thus, the aim of this study was to evaluate the nutrient bioavailability and growth performance of Nile tilapia-fed plant-based diets supplemented with graded levels of phytase and subjected to commercially intensive rearing conditions.

## 2. Material and Methods

The experiment was conducted in a commercial fish farm located in the Paranapanema River dam (Palmital, SP, Brazil) and was divided into two phases. During phase I, the effects of graded phytase supplementation levels on growth performance were evaluated. In phase II, a parcel of animals from phase I were subjected to a digestibility trial to evaluate the effects of phytase supplementation on the bioavailability of dry matter, gross energy, crude protein, and ash. In both phases, fish were kept in floating net cages under commercially intensive rearing conditions.

### 2.1. Experimental Diets

The experimental diets were formulated to contain 28% digestible protein and 12.71 M kg^−1^ digestible energy, according to [18,19]. All the nutritional requirements of Nile tilapia were met with the exception of phosphorus, whose inclusion as dicalcium phosphate was intentionally reduced to produce phosphorus-deficient diets (Table 1). In addition to the phosphorus-deficient diets, a positive control diet with similar composition was formulated and supplemented with inorganic phosphorus from dicalcium phosphate to meet the nutritional requirement of Nile tilapia for this mineral. Microbial phytase (SP1002, F. Roche Vitamins Ltd., Basel, Switzerland) was incorporated into the diets at levels of 0, 500, 1000, 1500, and 2000 UF kg^−1^ according to previous studies [17,20]. In this study, one phytase unit (FTU) was defined as the activity of the enzyme that liberates 1 μmol of inorganic orthophosphate (Pi) min^−1^ at pH 5.5, 37 °C, at a substrate (sodium phytate) concentration of 5.1 mmol/l [21].

The ingredients were grounded, sieved (1.0 mm), mixed, and extruded into a 4.0 mm pellet according to industry practices (Bernardino de Campos-Bercamp, SP, Brazil). After extrusion, the pellets were dried at 90ºC, cooled, and bagged until further use. At the fish farm, the pellets were top coated with phytase according to the concentrations previously described. Briefly, phytase was weighed, diluted in water, and sprayed over the pellets. A concrete mixer was utilized to homogenize the mixture. Then, all diets (with and without phytase) were top coated with 2% of soybean oil (considered in the feed formulation) and further homogenized.

### 2.2. Phase I-Growth Trial

All-male Nile tilapia juveniles (*n* = 6300, 57.48 ± 1.04 g) were randomly stocked into 42 floating cages (1.0 m^3^ and 19 mm mesh net, 150 fish/tank) in an average depth of 10 m with seven replicates per experimental diet in a completely randomized design.

Fish were fed three times a day (7:00 a.m., 12:00 a.m., 4:00 p.m.) for 97 days based on their biomass, as shown in Table 2. The corresponding feed intake (FI) was recorded daily. Water quality parameters were measured using a YSI 556^®^ probe and the average values ± SD were recorded as follows: temperature = 26.5 ± 0.5 °C; pH = 7.14 ± 0.28, and dissolved oxygen = 6.20 ± 0.91 mg L^−1^.

Before starting the feeding trial (Phase I), the initial number of fish (IN) was counted, and the tank’s initial biomass (IB) was measured. At the end of this phase, the final tank biomass (FB) and the final number of fish (FN) were recorded. Weight gain (WG; g/tank), feed conversion rate (FCR), and daily weight gain (DWG; g/fish) were calculated as follows: WG (%) = (FB − IB) × 100/IB; FCR = FI/(FB − IB); DWG = [(FB − IB)/FN]/97. The survival rate over the experimental period was calculated as follows: SUR (%) = 100 ((IN − FN)/IN).

### 2.3. Phase II-Digestibility Assessment

During this experimental phase, Nile tilapia (*n* = 900, 500 ± 10 g) were allocated in a feeding system composed of 6 floating net cages (1.0 m^3^, 19 mm mesh net, 150 fish/tank) at the same fish farm where the growth trial was conducted. The diets utilized in the digestibility trial were manufactured to contain the same nutritional value as those fed to fish during the growth trial. Chromic oxide (Cr_2_O_3_) was used as an inert digestibility marker (0.1% of dry weight) in all diets.

Fish were fed the respective experimental diets during an adaptation period of ten days. After that period, fish were fed three times a day (7:00 a.m., 12:00 a.m., and 4:00 p.m.) based on their biomass.

In order to enhance the fecal material collection, six conical-shaped net feces collectors were developed (1.5 m^3^, 1.6 mm mesh net) and used to sheath the feeding net cages overnight. At 5 pm, the feces collectors were attached to the feeding system, being detached at 6 am of the subsequent day for sample collection. Sampling was carried out for three days (1 replicate for each treatment a day). The feces samples were oven-dried (50 °C, 24 h), grounded and stored at −20 °C until further analysis.

### 2.4. Chemical Analyses

The chemical composition of both feces and diets was determined following the standard protocols of A.O.A.C. [22]. Dry matter (DM) was determined by oven-drying at 105 °C for 6 h. The Kjeldahl methodology was used to quantify the nitrogen content, and crude protein (CP) and then calculated as %N × 6.25. Gross energy (GE) was assessed using an adiabatic bomb calorimeter (C200, IKA, Staufen, BW, Germany) following the manufacturer instructions. Ash content was determined by burning samples at 550 °C for 6 h. The apparent digestibility coefficients for dry matter (ADC_DM_), gross energy (ADC_GE_), and crude protein (ADC_CP_), were calculated according to [23]:

(1) $${\mathrm{ADC}}_{\left(N\right)}=100-\left[100\left(\frac{{\%\mathrm{Cr}}_{2}0_{3D}}{\%\mathrm{Cr}0_{3F}}\right)\times \left(\frac{{\%N}_{F}}{{\%N}_{D}}\right)\right]$$

in which ADC_N_ = apparent digestibility coefficients of a nutrient, %Cr_2_O_3D_ = % chromic oxide in diet, %Cr_2_O_3_f = % chromic oxide in feces, %Nf = % of nutrients (or kJ g^−1^ gross energy) in feces and %Nd = % nutrient diet (or kJ g^−1^ gross energy) in the diet.

### 2.5. Statistical Analysis

Data obtained from the growth performance and digestibility trials were tested for homogeneity and normality using Browne-Forsythe and Shapiro-Wilk test, respectively. After that, the data from both trials were subjected to one-way analysis of variance (ANOVA) with the assistance of SAS statistical program (2002) to verify if the dietary supplementation levels of phytase resulted in significant effects (*p* < 0.05). If significant effects were observed via ANOVA, Tukey’s test was applied to compare the means (*p* < 0.05)

Additionally, after ANOVA evaluation daily weight gain and feed conversion ratio data were submitted to a second-order polynomial model (y = ax^2^ + bx + c) to determine the optimal dietary level of phytase.

## 3. Results

### 3.1. Growth Performance

**Figure 1:**
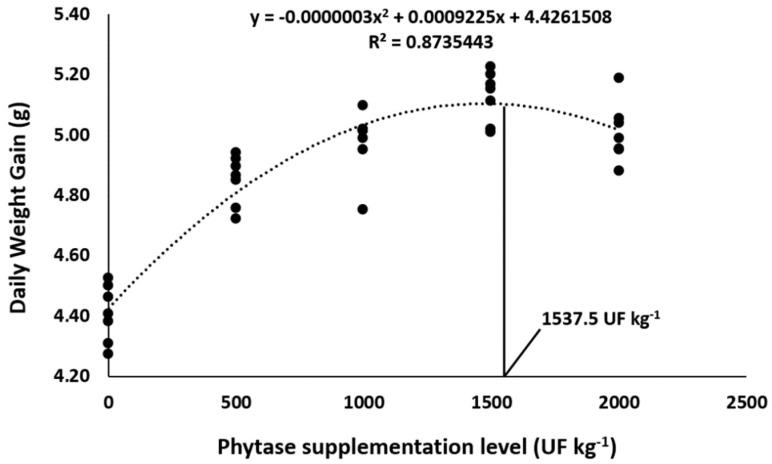
Daily weight gain of Nile tilapia fed diets supplemented with graded levels of phytase. The black points represent the seven replicates of each dietary treatment. Lines were used to mark the optimum supplementation level of phytase determined by the quadratic regression.

**Figure 2:**
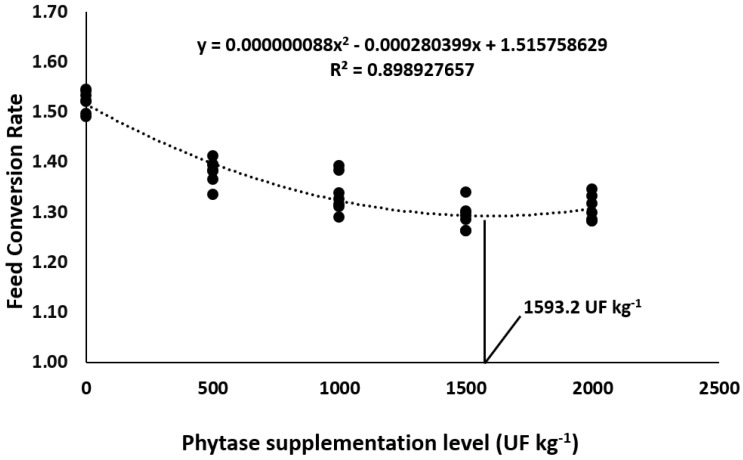
Feed conversion rate of Nile tilapia fed diets supplemented with graded levels of phytase.

Overall, phytase dietary supplementation in Nile tilapia diets improved the growth performance parameters in comparison to control diets (Table 3). Final weight, final biomass, weight gain, and daily weight gain were affected the most by 1500 and 2000 UF Kg^−1^, which resulted in the highest means compared to the other supplementation levels (*p* < 0.05). Supplementing 1500 and 2000 UF kg^−1^ promoted the lowest feed conversion rates compared to the other supplementation levels (*p* < 0.05). Quadratic polynomial regressions indicated 1537.5 and 1593.2 UF kg^−1^ as the optimum dietary levels for daily weight gain (Figure 1) and feed conversion rate (Figure 2), respectively. Lastly, the addition of phytase in Nile tilapia diets had no effect on the survival rate in this study.

### 3.2. Digestibility Assessment

A significant effect of phytase supplementation on ADC values was found in this study (Table 4). Supplementing 2000 UF kg^−1^ resulted in the highest ADC_DM_ value, followed by the other levels and both control diets, which did not statistically differ (*p* < 0.05). The ADC_CE_ values were most affected by 2000 UF kg^−1^ level, determining the higher ADC_CE_ (*p* < 0.05), followed by the positive control, which did not differ from 500, 1500 UF kg^−1^, and negative control. The 2000 UF kg^−1^ level also promoted the highest ADC_CP_ (*p* < 0.05) followed by the positive control, while the other levels and negative control did not statistically differ. ADC_ASH_ was most affected by the 2000 UF kg^−1^ level in comparison to other phytase dietary levels and control diets (*p* < 0.05).

## 4. Discussion

Phytase supplementation benefits on fish growth and nutrient bioavailability have been described well in previous reports. However, to the authors’ knowledge, there are no studies evaluating the effects of phytase on Nile tilapia’s productive performance and nutrient digestibility under commercially intensive rearing conditions. Indeed, Nile tilapia-fed phytase-supplemented diets from 1500 to 2000 UF kg^−1^ presented better growth when compared to the control groups. These findings may be associated with phytate sequential hydrolysis catalyzed by phytase, which reduces the anti-nutritional effects of phytate. Phytate can bind to Zn, Ca, trace minerals, cation groups of amino acids, lipids, protein, and starch reducing their availability in animal diets [2]. This molecule can also bind to enzymes including alpha-amylase, pepsin, and trypsin, inhibiting their activity, which considerably worsens protein digestibility, availability of amino acids, and energy [24].

The optimum supplementation levels for growth performance of Nile tilapia juveniles found in this study are higher than those established by previous reports which ranged from 700 to 1000 UF kg^−1^ [20,25]; but are still in accordance with those found by [17,26], ranging from 1250 to 2000 UF kg^−1^, respectively, under laboratory conditions. This divergence might be associated with several factors such as enzyme source, phytase inclusion method, dietary content of phytate, and the commercially intensive rearing conditions. It is well known that the microbial source of phytase impacts its efficiency, thus different supplementation levels may achieve similar results depending on the enzyme intrinsic characteristics. The supplementation method and feed processing also exert influence on phytase efficiency. When phytase is added prior to feed processing, its efficiency is usually reduced, especially if feed is processed via extrusion as observed in a previous study [27].

The type of plant ingredients used in the feed formula also influence phytase effects. That is because phytate content of a given diet vary depending on the plant ingredient used, directly influencing phytase activity. The content of phytate in plant ingredients considerably varies, as observed by [28]. Therefore, the enzyme source and inclusion method, amount and type of plant ingredients present in the experimental diets could also affect phytase efficiency. Another important aspect is enzyme lixiviation. Under commercially intensive rearing conditions, the water influx is substantially higher if compared to a traditional laboratory RAS system. This could contribute to the need of higher phytase supplementation levels to result in similar growth performance gains obtained under laboratory conditions.

Negative effects of dietary phytate content were also observed in the digestibility assessment results. Overall, the supplementation of phytase at intermediary levels (500, 1000, and 1500 UF kg^−1^) showed a limited impact on ADC values for dry matter, crude protein, energy, and ash when compared to negative and positive control diets. Substantial effects of phytase in the evaluated ADC values were observed mainly at the highest level of supplementation (2000 UF kg^−1^). The reduced ADC of nutrient and energy findings could be attributed to the chelating capacity of phytate that binds itself to different dietary components and enzymes, reducing the digestibility of nutrients, energy, and the activity of specific enzymes, as well as the field conditions potential effects previously discussed in this manuscript.

Regarding ADC_CP_ values, the results of the present study show that phytase supplementation up to 2000 UF kg^−1^ can improve crude protein digestibility. In fact, there is controversy about the effects of phytase on ADC_CP_ values in fish nutrition studies. It was verified that phytase supplementation at levels ranging from 660 to 4500 UF kg^−1^ in juvenile Nile tilapia diets had no positive effects on ADC_CP_ values in comparison to the control diet [14,29]. Conversely, ADC_CP_ was enhanced by phytase supplementation up to 1000 UF kg^−1^ for the same fish species [15]. It was verified that phytase inclusion enhanced crude protein apparent digestibility compared to the control diet at 500, 1500, and 3000 UF kg^−1^ [20] and 500, 1000, 2000, and 4000 UF kg^−1^ [17]. In addition, 1000 UF kg^−1^ increased ADC_CP_ values in diets with soybean as the main protein source [9].

The effects of phytase on crude energy digestibility showed a similar trend as ADC_CP_. Only the 2000 UF kg^−1^ dietary level was capable of increasing the ADC_CE_ in comparison to the control diets. Those results could be related to the phytate content of plant ingredients, which are complexes with starch and enzymes, reducing the availability of energy sources and the activity of important enzymes. In fact, [30] verified that phytate reduced the digestibility of starch by 28% during an in vitro assay. This finding is associated with phytate interaction with alpha-amylase protein [24] and Ca, which is a known cofactor of the later enzyme [31]. The benefits of phytase on energy availability were also observed by [9,14,29] supplementing 600, 1000, and 1500 UF kg^−1^ in juvenile Nile tilapia diets, respectively. As to ADC_DM_, only the highest level of phytase inclusion resulted in an improvement. Similarly to our findings, [29] observed that including 1500 UF kg^−1^ in plant-based diets for juvenile Nile tilapia resulted in an increase in ADC_DM_ values. Nevertheless, [17] verified no influence of phytase on dry matter digestibility for juveniles of the same species.

ADC_ASH_ observed in this study was positively affected by phytase supplementation with a peak at 2000 UF kg^−1^, promoting an increase of 40% and 42% compared to the positive and negative control diets respectively. Dietary phytase supplementation in plant-based fish diets considerably enhances the availability of phosphorus, calcium, and other minerals [2]. The apparent digestibility coefficient of Ash represents the bioavailability of dietary inorganic mineral content. Therefore, due to the above-mentioned effects of phytase in phytate hydrolysis, the supplementation of this enzyme also affects ADC_Ash_ values. Such effects were observed in recent studies carried out under laboratory conditions. It was verified that supplementing 660 UF kg^−1^ in juvenile Nile tilapia plant-based diets increased ADC_Ash_ by about 11% compared to the control diet [14]. In another study [32], the same author observed an increase of 18% in ADC_Ash_ of Nile tilapia diets containing 1000 UF kg^−1^ in comparison to the control group. Positive effects of different levels of phytase supplementation on ADC_Ash_ of plant-based diets are usually observed in studies with Nile tilapia and other fish species [17,32,33]

Differences between previous reports were expected since this study was conducted under commercially intensive rearing conditions. Under these conditions, there are several external parameters that could influence phytase efficiencies such as temperature, pH, and enzyme lixiviation.

## 5. Conclusions

Based on the polynomial regression analysis of the feed conversion rate, the recommended level of phytase supplementation in diets for juvenile Nile tilapia under commercially-intensive rearing conditions was estimated to be 1593.2 UF kg^−1^. Overall, phytase supplementation positively affected Nile tilapia growth performance and nutrient digestibility. This study provides scientific evidence that findings of digestibility and growth performance trials conducted under laboratory conditions are consistent with the effects of phytase supplemented in juvenile Nile tilapia diets under commercially intensive rearing conditions. Considering these results, phytase supplementation may enhance tilapia farming sustainability by optimizing nutrient digestibility and reducing the need for inorganic mineral sources.

## Figures and Tables

**Table 1:** Formulation and proximate composition of experimental diets containing graded levels of phytase supplementation.

Ingredients (%)	Phytase Supplementation Levels					
	PC ^5^	NC ^6^	500 UF kg^−1^	1000 UF kg^−1^	1500 UF kg^−1^	2000 UF kg^−1^
Soybean meal	59.88	59.78	59.78	59.78	59.78	59.78
Corn	23.5	24.03	24.03	24.03	24.03	24.03
Rice bran	12.00	12.00	12.00	24.03	12.00	12.00
Soybean oil	1.00	1.00	1.00	1.00	1.00	1.00
DL-methionine	0.28	0.28	0.28	0.28	0.28	0.28
Threonine	0.22	0.22	0.22	0.22	0.22	0.22
Dicalcium phosphate	1.90	0.70	0.70	0.70	0.70	0.70
Limestone	-	0.77	0.77	0.77	0.77	0.77
Mold-zap ^1^	0.1	0.1	0.1	0.1	0.1	0.1
Banox ^2^	0.02	0.02	0.02	0.02	0.02	0.02
Vit/Min Premix ^3^	0.5	0.5	0.5	0.5	0.5	0.5
NaCl	0.5	0.5	0.5	0.5	0.5	0.5
Chromium oxide ^4^	0.1	0.1	0.1	0.1	0.1	0.1
Total (%)	100	100	100	100	100	100
**Proximate analysis**						
Gross Energy (MJ)	17.60	17.48	17.36	17.15	17.33	17.44
Crude Protein (%)	35.12	34.69	34.79	35.69	34.75	34.25
Dry Matter (%)	91.43	90.36	89.64	89.52	90.87	90.25
Ash (%)	8.60	9.10	9.43	9.35	9.37	9.20

^1^ Mold-Zap^®^—Antifunfic, Alltech Agroindustrial Ltd.a, São Paulo, Brazil. ^2^ Banox—Antioxidant, Alltech Agroindustrial Ltd.a, São Paulo, Brazil. ^3^ Premix—Vitamin and mineral premix, Mogiana Alimentos (as active matter per kg): Vit. A: 16.000 UI; Vit. B1: 32 mg; Vit. B2: 32 mg; Vit. B6: 32 mg; Vit. B12: 32 mcg; Folic acid: 10 mg; Vit. E: 250 UI; Vit. K3: 30 mg; Niacin: 170 mg; Biotin: 10 mg; Vit. D3: 4.500 UI; Vit. C: 325 mg; Choline: 1000 mg; Manganese: 50 mg; Selenium: 0,70 mg; Iron: 150 mg; Copper: 20 mg; Iodine: 1 mg; Cobalt: 0.50 mg; Ca pantothenate: 80 mg; Zinc: 150 mg. ^4^ Cr_2_O_3_—Vetec Química Fina Ltd.a., Duque de Caxias, RJ, Brazil. ^5^ PC—positive control. ^6^ NC—negative control. UF-Phytase Units.

**Table 2:** Daily feed rations offered according to fish biomass.

Fish Weight Interval (g)		Daily Feed as Biomass Weight (%)
80	150	5
150	300	4
300	400	3.5
400	600	3

**Table 3:** Growth performance of Nile tilapia fed diets supplemented graded levels of phytase.

Parameters	Phytase Supplementation Levels						
	NC	PC	500 UF kg^−1^	1000 UF kg^−1^	1500 UF kg^−1^	2000 UF kg^−1^	*p* Value
IW(g)	57.20 ± 1.08	57.05 ± 1.13	57.94 ± 1.28	57.33 ± 0.83	57.75 ± 1.08	57.05 ± 1.13	0.548
FW(g)	491.35 ± 9.27 ^d^	530.59 ± 5.54 ^c^	531.56 ± 9.29 ^bc^	544.18 ± 10.95 ^abc^	557.93 ± 9.01 ^a^	546.07 ± 10.36 ^ab^	0.000
IB(g)	8580 ± 162	8597 ± 158	8691 ± 192	8600 ± 125	8663 ± 161	8597 ± 158	0.676
FB(g)	69,013 ± 829 ^c^	75,791 ± 1226 ^b^	75,237 ± 1233 ^b^	77,429 ± 2 052 ^ab^	79,700 ± 1522 ^a^	79,011 ± 1395 ^a^	0.000
WG(%)	750.08 ± 25.38 ^d^	830.37 ^c^ ± 16.80 ^c^	829.46 ± 17.17 ^c^	839.45 ± 23.41 ^bc^	873.17 ± 11.76 ^a^	845.69 ± 19.15 ^ab^	0.000
DWG(g)	4.43 ± 0.08 ^d^	4.85 ± 0.05 ^c^	4.85 ± 0.08 ^c^	4.98 ± 0.11 ^bc^	5.13 ± 0.08 ^a^	5.01 ± 0.10 ^ab^	0.000
FCR(g)	1.52 ± 0.02 ^a^	1.37 ± 0.03 ^bc^	1.38 ± 0.02 ^b^	1.34 ± 0.03 ^cd^	1.29 ± 0.03 ^d^	1.31 ± 0.03 ^d^	0.000
Survival(%)	95.24 ± 1.86	93.90 ± 2.09	94.38 ± 2.07	94.86 ± 1.71	95.24 ± 1.41	96.48 ± 1.71	0.146

Data shown as mean ± SD of seven replicates. Means within the same row that share a common letter do not differ statistically (*p* < 0.05). IW—initial weight; FW—final weight; IB—initial biomass; FB—final biomass; WG—weight gain; DWG—daily weight gain; FCR—feed conversion rate; UF—phytase units.

**Table 4:** Apparent digestibility coefficient (ADC) of Nile tilapia-fed diets supplemented with graded levels of phytase.

ADC (%)	Phytase Supplementation Levels						
	PC	NC	500 UF kg^−1^	1000 UF kg^−1^	1500 UF kg^−1^	2000 UF kg^−1^	*p* Value
DM	76.82 ± 0.58 ^b^	74.82 ± 1.85 ^b^	77.04 ± 2.71 ^b^	74.17 ± 2.25 ^b^	76.24 ± 0.61 ^b^	89.06 ± 1.45 ^a^	0.000
CE	83.97 ± 0.49 ^b^	82.5 ± 1.81 ^bc^	83.36 ± 2.34 ^bc^	79.46 ± 1.82 ^c^	80.98 ± 0.76 ^bc^	92.35 ± 0.87 ^a^	0.000
CP	94.46 ±0.11 ^b^	92.66 ± 0.89 ^c^	93.61 ± 0.44 ^bc^	92.78 ± 0.74 ^c^	92.16 ± 0.25 ^c^	97.26 ± 0.35 ^a^	0.000
ASH	29.49 ± 3.54 ^d^	27.97 ± 7.52 ^cd^	42.25 ± 2.79 ^bc^	46.17 ± 5.61 ^b^	46.65 ± 2.15 ^b^	69.71 ± 6.04 ^a^	0.000

Data shown as means ± SD of three replicates. Means within the same row that share a common letter do not differ statistically (*p* < 0.05). DM—dry matter; CE—crude energy; CP—crude protein; PC—positive control; NC—negative control; UF—phytase units.

## Data Availability

Data for the feeding and digestibility trial can be provided upon reasonable request to the corresponding authors.
